# Influence of FTO variants on obesity, inflammation and cardiovascular disease risk biomarkers in Spanish children: a case–control multicentre study

**DOI:** 10.1186/1471-2350-14-123

**Published:** 2013-12-01

**Authors:** Josune Olza, Azahara I Ruperez, Mercedes Gil-Campos, Rosaura Leis, Dietmar Fernandez-Orth, Rafael Tojo, Ramon Cañete, Angel Gil, Concepcion M Aguilera

**Affiliations:** 1 Department of Biochemistry and Molecular Biology II, Faculty of Pharmacy Institute of Nutrition and Food Technology, University of Granada, Granada, Spain; 2 Paediatric Research and Metabolism Unit, Reina Sofía University Hospital, Maimonides Institute for Biomedical Research (IMIBIC), Córdoba, Spain; 3 Unit of Investigation in Nutrition, Growth and Human Development of Galicia, Paediatric Department, Clinic University Hospital of Santiago, University of Santiago de Compostela, Galicia, Spain; 4 Progenika Biopharma S.A, Building 504, Zamudio Technologic Park. Derio, Bizkaia, Spain

**Keywords:** FTO, Genetic polymorphism, Obesity, Child, Inflammation, CVD

## Abstract

**Background:**

Variants in the *FTO* gene have been associated with obesity in children, but this association has not been shown with other biomarkers. We assessed the association of 52 *FTO* polymorphisms, spanning the whole gene, with obesity and estimated the influence of these polymorphisms on anthropometric, clinical and metabolic parameters as well as inflammation and cardiovascular disease (CVD) risk biomarkers among Spanish children.

**Methods:**

A multicentre case–control study was conducted in 534 children (292 obese and 242 with normal-BMI). Anthropometric, clinical, metabolic, inflammation and CVD risk markers were compared using the Student’s t-test for unpaired samples. The genotype relative risk was assessed by comparing the obese and normal-BMI group, calculating the odds ratio. The association of each SNP with phenotypic parameters was analysed using either logistic or linear regression analysis.

**Results:**

All anthropometric, clinical and metabolic factors as well as inflammatory and CVD risk biomarkers were higher in the obese than in the normal-BMI group, except adiponectin and HDL-c that were lower, and glucose, LDL-c, and metalloproteinase-9 that did not show difference. Four polymorphisms (rs9935401, rs9939609, rs9928094 and rs9930333) were positively associated with obesity and in linkage disequilibrium between each other; the haplotype including the risk alleles of these polymorphisms showed a high risk for obesity. The rs8061518 was negatively associated with obesity and the haplotype including this SNP and rs3826169, rs17818902 and rs7190053 showed a decreased risk for obesity. Additionally, the rs8061518 was associated with weight, diastolic blood pressure, insulin, homeostatic model assessment of insulin resistance, leptin, and active plasminogen inhibitor activator-1 after sex and age adjustment; however, after an additional BMI adjustment, this polymorphism remained associated only with leptin.

**Conclusions:**

We validated the previous reported association of genetic variability in intron 1 of the *FTO* gene with the risk of obesity and found no association with other related traits in this region of the gene. We have observed strong statistical evidence for an association of rs8061518 in intron 3 of the gene with decreased risk of obesity and low concentration of leptin.

## Background

Obesity is described as a disease with genetic and environmental influence that may lead to several comorbidities such as type 2 diabetes, cardiovascular diseases (CVD), metabolic syndrome (MS) and respiratory abnormalities among others [1,2]. Since the discovery of its association with variants in intron 1 of the *fat mass and obesity associated* (*FTO)* gene [3-5], many studies have been conducted to replicate this finding, to identify new variants, to clarify the biological function of the protein encoded and to explain its role in the origin and/or development of obesity [6]. *FTO* was initially associated with type 2 diabetes mellitus in European descent population [3]; however, simultaneous studies showed that the association was mediated by its effect on obesity [4,5]. This association with obesity also has been observed in different populations as Europeans [4,5,7-9], Asians [10,11], African Americans [12,13] and Hispanic Americans [13]. Among the identified polymorphisms, the rs9939609 is one of the most strongly associated with common obesity [10,14,15]; however, several other single nucleotide polymorphism (SNP)s in the first intron of the *FTO* gene that are in linkage disequilibrium (LD) with rs9939609 showed similar effects on both childhood [4,7,16] and adult obesity [3,4].

It is common to observe clinical, metabolic and CVD risk factors in many, but not all obese children. However, few studies include a large number of biomarkers of inflammation and CVD risk in the paediatric population and compare the levels of these markers with the presence or absence of genetic variants. For this reason, we assessed the association of 52 SNPs spanning the *FTO* gene, with obesity in Spanish children; we also estimated the influence of these SNPs in anthropometric, clinical and metabolic parameters as well as inflammatory and CVD risk markers.

## Methods

### Study design

The present study was designed as a case–control multicentre study in children. We recruited 534 children, 292 classified as obese according to body mass index (BMI), using the age- and sex-specific cut-off points of Cole *et al.*[17] (149 boys and 143 girls), and 242 as normal-BMI (135 boys and 107 girls). The children were prepubertal and pubertal aged 6–15 years and were recruited in two cities of Spain (Cordoba and Santiago de Compostela) from primary care centres and schools. Inclusion criterions were European descent and the absence of congenital metabolic diseases. Exclusion criterions were non-European descent, the presence of congenital metabolic diseases, for example, diabetes or hyperlipidaemia, or undernutrition and the use of medication that alters blood pressure (BP) and glucose or lipid metabolism. After assessments made on a first visit to the school or primary care centre, the parents of children fulfilling the inclusion criteria were invited to take the children to the paediatrics unit of the participating hospitals for a clinical examination.

### Ethics statements

The protocol was performed in accordance with the *Declaration of Helsinki (Edinburgh 2000 revised*) and following the recommendations of the *Good Clinical Practice of the CEE (Document 111/3976/88 July 1990)* and the in force law of the Spanish regulation, that regulates *Clinical Trials in human beings (RD 223/04 about Clinical Trials).* The Ethics Committee on Human Research of the University of Granada, the Ethics Committee of the Reina Sofía University Hospital of Cordoba and the Bioethics Committee of the University of Santiago de Compostela approved it. Written informed consent was obtained from parents of children, and all children gave their assent.

### Anthropometric and biochemical measurements

Anthropometric measurements were taken by a single examiner with the children barefoot and in their underwear. Body weight (kg) and height (cm) were measured using standardized procedures, and waist circumference (WC) was measured midway between the lowest rib and the superior border of the iliac crest on standing subjects, using an inelastic measuring tape at the end of normal expiration. BMI was calculated as weight (kg) divided by the square of the height (m^2^). BP was measured three times by the same examiner, using a mercury sphygmomanometer and following international recommendations. Blood samples were drawn *via* the antecubital vein after the patient had fasted overnight. Insulin resistance was calculated by means of the homeostatic model assessment of insulin resistance (HOMA-IR). General biochemical analyses were performed at the participating University Hospital Laboratories following internationally accepted quality control protocols.

### Cardiovascular and inflammatory biomarkers

Three different LINCOplex^TM^ kits of human monoclonal antibodies (Linco Research, St. Charles, MO) were used on a Luminex® 200^TM^ System (Luminex Corporation, Austin, TX) to determine: 1) adiponectin (coefficient of variation [CV]: 7.9%), resistin (CV: 6.0%) and active plasminogen activator inhibitor-1 (PAI-1) (CV: 6.6%) (Cat. HADK1-61 K-A); 2) interleukin (IL)-6 (CV: 7.8%), IL-8 (CV: 7.9%), leptin (CV: 7.9%) and tumour necrosis factor alpha (TNF-α) (CV: 7.8%) (Cat. HADK2-61 K-B); and 3) soluble intercellular adhesion molecule 1 (sICAM-1) (CV: 7.9%), soluble endothelial selectin (sE-selectin) (CV: 11.2%), myeloperoxidase (MPO) (CV: 12.3%), matrix metalloproteinase 9 (MMP-9) (CV: 6.8%) and total PAI-1 (CV: 11.8%) (Cat. HCVD1-67 AK). C reactive protein (CRP) (CV: 4%) was determined with a particle-enhanced turbidimetric immunoassay (PETIA) (Dade Behring Inc., Deerfield, IL).

### DNA isolation and genotyping

Genomic DNA was extracted from peripheral white blood cells (buffy coat) using the QIAamp Blood kit (Qiagen, Valencia, CA) according to the manufacturer instructions. 52 SNPs in the *FTO* gene were selected. SNPs eligibility was based on their location, first selecting every missense variation and then others located in the promoter, 3′UTR and 5′UTR regions with a minor allele frequency (MAF) higher than 0.05 in the European descent population from the NCBI database and a minimum pair wise LD of r^2^ = 0.8 for the selection of TagSNPs from the HapMap. In addition, SNPs described to be positively associated with obesity in relevant publications were also included. Genotyping was performed by the Illumina GoldenGate Assay (Illumina Inc., San Diego, CA) on 96-well format Sentrix® arrays. Two hundred fifty nanograms of DNA samples were used per assay. Genotyping of the 52 SNPs resulted in a success rate >97%. Five SNPs (rs1125337, rs9929152, rs16952649, rs8053966 and rs8054364), which deviated from Hardy-Weinberg equilibrium in the normal-BMI group (p < 0.05) were excluded from the analysis (Additional file 1: Table S1). Because our population proceeds from two different cities of Spain, we performed a meta-analysis with the two populations as independent groups to avoid problems like population stratification or genotyping batch effect. The result of this analysis (p values of Q Cochrane: rs9939609 Q = 0.967, rs9935401 Q = 0.888, rs9930333 Q = 0.795, rs9928094 Q = 0.867 and rs8061518 Q = 0.926) indicate little detectable heterogeneity for the two considered populations of the study.

### Statistical analysis

All continuous variables were expressed as mean ± SEM. After checking for skewness and kurtosis, insulin, HOMA-IR, total cholesterol, MMP-9 and total PAI-1 were normalized using logarithmic transformation. Homogeneity of variances was estimated using the Levene test. Comparisons between obese and normal-BMI children variables were assessed using the Student’s t-test for unpaired samples using SPSS (version 15.0.1. Chicago, IL). Genotype and allele frequencies were calculated for cases and controls. The genotypic relative risk was assessed by comparing the obese and normal-BMI group, calculating the odds ratio (OR) and 95% confidence interval (CI), using logistic regression analysis under an additive model adjusted by sex and age with a Bonferroni correction using the SNPassoc package from R software [18]. Logistic or linear regressions analysis in the entire population was performed under an additive model to estimate the associations of each SNP with phenotypic parameters related to obesity as well as biomarkers of CVD risk and inflammation, adjusted by sex and age using the PLINK software. Haplotype analysis involved the use of Haploview version 4.2 software and haplo.stats package from R software. Logistic regression analyses was performed to assess the association of the studied haplotypes with obesity; the model generated computes a χ^2^ to estimate the p value that test the fit of the model. This p explains the variation in the phenotype compared to a model with no effect. Significance was considered at the level of p < 0.05.

## Results

### General characteristics of the population

Table 1 shows the anthropometric, clinical and metabolic characteristics of obese and normal-BMI subjects. Weight, height, BMI, BMI z-score and WC were significantly higher in the obese compared with the normal-BMI children. Systolic and diastolic BP, as well as plasma triacylglycerols, apolipoprotein B (ApoB), insulin, and HOMA-IR, were higher in obese children, whereas plasmatic total cholesterol, high-density lipoprotein-cholesterol (HDL-c) and apolipoprotein A1 (ApoA1) were lower in obese children. Fasting plasma glucose and low-density lipoprotein-cholesterol (LDL-c) concentrations did not show differences between groups. Plasmatic resistin and leptin concentrations were significantly higher in obese than in normal-BMI subjects, by contrast decreased levels of adiponectin were found in obese children. Inflammatory and CVD risk biomarkers were different between groups. CRP, IL-6, IL-8 and TNF-α were significantly higher in the obese group compared with those in the normal-BMI group. Likewise, plasmatic sICAM-1, sE-selectin, MPO and active and total PAI-1 were higher in the obese group, whereas MMP-9 levels did not show difference between groups.

**Table 1:** Anthropometric, clinical and biochemical parameters of the children

	**Normal-weight**	**Obese**	**P**
n	242	292	
**Anthropometric factors**			
Sex (M/F)	135/107	149/143	0.273
Age (y)	9.73 ± 0.2	9.43 ± 0.2	0.172
Weight (kg)	32.9 ± 0.7	55.9 ± 1.0	**<0.001**
Height (m)	1.37 ± 0.01	1.41 ± 0.01	**0.001**
BMI (kg/m^2^)	17.14 ± 0.13	27.56 ± 0.24	**<0.001**
BMI z-score	−0.17 ± 0.04	3.49 ± 0.08	**<0.001**
Waist circumference (cm)	60.2 ± 0.5	84.0 ± 0.9	**<0.001**
**Clinical and metabolic biomarkers**			
Systolic BP (mm Hg)	97 ± 1	111 ± 1	**<0.001**
Diastolic BP (mm Hg)	60 ± 1	69 ± 1	**<0.001**
Glucose (mg/dl)	84 ± 0	85 ± 1	0.772
Insulin (mU/l)	5.95 ± 0.23	11.46 ± 0.51	**<0.001**
HOMA-IR	1.26 ± 0.05	2.43 ± 0.12	**<0.001**
Triacylglycerols (mg/dl)	55 ± 1	75 ± 2	**<0.001**
ApoAI (mg/dl)	150 ± 2	132 ± 1	**<0.001**
ApoB (mg/dl)	67 ± 1	71 ± 1	**0.008**
Cholesterol (mg/dl)	171 ± 2	165 ± 2	**0.012**
HDL-c (mg/dl)	64 ± 1	51 ± 1	**<0.001**
LDL-c (mg/dl)	94 ± 2	97 ± 1	0.144
Adiponectin (mg/l)	28.28 ± 0.77	22.57 ± 0.66	**<0.001**
Resistin (μg/l)	9.65 ± 0.33	11.71 ± 0.34	**<0.001**
Leptin (μg/l)	4.30 ± 0.26	22.91 ± 0.86	**<0.001**
**Inflammatory biomarkers**			
C-reactive protein (mg/l)	0.48 ± 0.07	1.99 ± 0.16	**<0.001**
Interleukin 6 (ng/l)	4.50 ± 0.53	6.95 ± 0.75	**0.012**
Interleukin 8 (ng/l)	1.57 ± 0.11	2.16 ± 0.15	**0.003**
TNF-α (ng/l)	3.06 ± 0.11	4.01 ± 0.13	**<0.001**
**Cardiovascular disease risk biomarkers**			
MMP-9 (μg/l)	79.99 ± 3.15	88.42 ± 3.90	0.636
MPO (μg/l)	13.10 ± 1.16	21.51 ±1.70	**<0.001**
sE selectin (μg/l)	22.93 ± 0.76	31.26 ± 1.05	**<0.001**
sICAM-1 (mg/l)	0.153 ± 0.004	0.175 ± 0.005	**<0.001**
Active PAI-1 (μg/l)	5.16 ± 0.27	11.96 ± 0.57	**<0.001**
Total PAI-1 (μg/l)	18.95 ± 0.85	27.23 ± 1.01	**<0.001**

M: male; F: female; BMI: body mass index; BP: blood pressure; HOMA-IR: homeostasis model assessment for insulin resistance; Apo: apolipoprotein; HDL-c: high-density lipoprotein cholesterol; LDL-c: low-density lipoprotein cholesterol; TNF-α: tumor necrosis factor alpha; MMP-9: metalloproteinase-9; MPO: myeloperoxidase; sICAM-1: soluble intracellular adhesion molecule-1, PAI-1: plasminogen activator inhibitor.

### Association of SNPs with obesity

Five SNPs of the 52 included in the analysis were deviated from Hardy-Weinberg equilibrium in the normal-BMI group (p < 0.05). Those SNPs were excluded from the association analysis. Of the 47 SNPs, five were associated with obesity in children, adjusted by age and sex under an additive model after Bonferroni correction; the rs9935401, rs9939609, rs9928094 and rs9930333 located in the intron 1 were positively associated with obesity, whereas the rs8061518 in the intron 3 was negatively associated (Table 2).

**Table 2:** **Genotypic distribution of the *FTO *** **analysed polymorphisms and its association with obesity**

		**Case**			**Control**				**Minor Allele**				
**Polymorphism**	**Allele 1/allele 2**	**11**	**12**	**22**	**11**	**12**	**22**	**Minor allele**	***Case***	***Control***	**OR (95% CI)**	**P**	P corr*
**rs12445162**	G/A	253	39	0	203	37	2	A	0.066	0.085	0.63 (0.35 - 1.11)	0.108	1
**rs11075986**	C/G	249	42	1	200	38	4	G	0.075	0.097	1.07 (0.80 - 1.44)	0.630	1
**rs7203521**	A/G	110	143	39	90	116	36	G	0.381	0.388	0.93 (0.68 - 1.28)	0.667	1
**rs1861868**	A/G	85	155	52	63	118	60	G	0.453	0.494	0.78 (0.58 - 1.06)	0.108	1
**rs11643744**	A/G	157	117	18	123	104	14	G	0.268	0.278	0.86 (0.61 - 1.22)	0.404	1
**rs9928094**	**A/G**	**67**	**149**	**75**	**88**	**114**	**40**	**G**	**0.511**	**0.398**	**1.66 (1.22 - 2.24)**	**0.0009**	**0.018**
**rs9930333**	**T/G**	**67**	**150**	**74**	**87**	**112**	**41**	**G**	**0.511**	**0.398**	**1.64 (1.21 - 2.22)**	**0.001**	**0.023**
**rs10852521**	C/T	104	139	49	64	116	61	T	0.410	0.496	0.67 (0.49 - 0.90)	**0.008**	0.327
**rs7205986**	A/G	68	151	72	70	111	61	G	0.505	0.483	0.78 (0.46 - 1.34)	0.369	1
**rs9935401**	**G/A**	**72**	**150**	**69**	**95**	**111**	**33**	**A**	**0.419**	**0.370**	**1.76 (1.29 - 2.41)**	**0.0003**	**0.004**
**rs9939609**	**T/A**	**72**	**149**	**69**	**90**	**118**	**33**	**A**	**0.491**	**0.377**	**1.73 (1.26 - 2.37)**	**0.0006**	**0.009**
**rs3826169**	T/C	170	102	20	151	81	10	C	0.245	0.210	1.32 (0.92 - 1.89)	0.132	1
**rs8061518**	**A/G**	**159**	**107**	**22**	**95**	**108**	**32**	**G**	**0.262**	**0.365**	**0.56 (0.40 - 0.78)**	**0.0006**	**0.025**
**rs17818902**	T/G	162	107	22	145	84	13	G	0.261	0.227	1.19 (0.84 - 1.67)	0.329	1
**rs7190053**	C/T	190	87	13	165	67	7	T	0.196	0.172	1.21 (0.82 - 1.78)	0.338	1
**rs2111114**	A/G	175	107	10	151	81	9	G	0.219	0.203	1.19 (0.82 - 1.74)	0.362	1
**rs8044353**	G/A	259	31	1	219	23	0	A	0.056	0.047	1.21 (0.61 - 2.39)	0.589	1
**rs10521303**	C/A	91	151	48	64	133	42	A	0.400	0.436	0.84 (0.62 - 1.15)	0.284	1
**rs1558756**	C/T	86	152	54	69	132	41	T	0.441	0.443	1.14 (0.83 - 1.57)	0.421	1
**rs16952623**	T/C	215	72	3	181	58	2	C	0.134	0.128	0.98 (0.62 - 1.56)	0.930	1
**rs16952624**	C/T	289	1	0	242	0	0	T	0.002	0.000			1
**rs2111113**	G/C	247	45	0	208	30	2	C	0.079	0.068	1.00 (0.56 - 1.78)	0.996	1
**rs10852525**	G/A	229	59	1	194	41	5	A	0.105	0.105	0.89 (0.55 - 1.45)	0.641	1
**rs7194336**	G/T	98	144	50	63	131	48	T	0.418	0.470	0.74 (0.55 - 1.01)	0.053	1
**rs7203181**	C/A	142	111	39	101	109	32	A	0.329	0.356	0.88 (0.65 - 1.19)	0.412	1
**rs6499656**	G/C	218	67	7	189	48	5	C	0.140	0.121	1.30 (0.84 - 2.03)	0.237	1
**rs7191513**	G/A	98	145	49	82	110	50	A	0.418	0.441	0.96 (0.72 - 1.30)	0.805	1
**rs7194907**	T/C	81	153	57	65	105	67	C	0.458	0.509	0.87 (0.64 - 1.18)	0.370	1
**rs8056299**	A/G	104	140	47	77	116	49	G	0.402	0.441	0.84 (0.62 - 1.14)	0.255	1
**rs17225435**	A/G	225	61	6	194	46	2	G	0.122	0.104	1.51 (0.93 - 2.46)	0.093	1
**rs8049235**	G/A	100	156	34	91	108	43	A	0.383	0.396	1.01 (0.74 - 1.59)	0.930	1
**rs7199716**	C/T	101	146	44	91	116	35	T	0.402	0.384	0.91 (0.67 - 1.24)	0.547	1
**rs13334214**	C/T	178	102	6	155	71	13	T	0.198	0.204	1.07 (0.74 - 1.57)	0.710	1
**rs7194243**	C/T	180	93	19	147	87	8	T	0.227	0.214	0.82 (0.57 - 1.18)	0.295	1
**rs1136002**	T/C	135	130	27	109	111	22	C	0.316	0.320	0.90 (0.66 - 1.24)	0.529	1
**rs4784351**	A/G	154	113	20	122	96	16	G	0.268	0.275	0.92 (0.65 - 1.28)	0.612	1
**rs2540781**	C/A	225	54	13	190	47	5	A	0.136	0.119	0.87 (0.57 - 1.32)	0.507	1
**rs8049933**	C/T	231	52	7	195	41	4	T	0.115	0.103	0.83 (0.52 - 1.32)	0.425	1
**rs1558687**	C/T	169	107	15	134	92	14	T	0.233	0.250	0.94 (0.67 - 1.33)	0.738	1
**rs2075202**	T/G	277	14	1	231	11	0	G	0.026	0.023	0.69 (0.25 - 1.88)	0.469	1
**rs7200579**	C/G	257	33	2	216	25	1	G	0.065	0.055	0.91 (0.50 - 1.68)	0.772	1
**rs697771**	C/T	102	137	53	72	127	43	T	0.413	0.443	1.00 (0.74 - 1.36)	0.985	1
**rs12596638**	G/A	221	66	4	176	61	5	A	0.126	0.150	0.99 (0.64 - 1.53)	0.979	1
**rs1008400**	C/T	81	148	61	67	132	43	T	0.469	0.451	0.97 (0.71 - 1.32)	0.860	1
**rs12932373**	C/T	214	76	2	175	61	3	T	0.136	0.139	0.77 (0.49 - 1.21)	0.261	1
**rs2689248**	C/A	79	153	59	64	113	63	A	0.463	0.492	0.79 (0.59 - 1.07)	0.124	1
**rs17833492**	C/A	140	127	24	106	112	23	A	0.296	0.322	0.88 (0.63 - 1.22)	0.437	1

CI: confidence interval; OR: odds ratio. The OR was adjusted for age and sex under the additive model. *P values after Bonferroni correction. SNPs are ordered according to their genomic position.

### Association of SNPs with obesity-related traits

Table 3 shows the association of the rs9939609 with obesity relate-traits. MMP-9 was the only biomarker associated with the former SNP after adjustment for sex, age and BMI [β = 0.03 μg/l; 95% CI: (0.0004, 0.0690] (p = 0.048). The analyses of the other SNPs positive associated with obesity are included in the Additional file 1: Tables S2–S4.

**Table 3:** Association of rs9939609 with anthropometric, clinical, inflammation and CVD risk biomarkers in children

**Biomarkers**	**TT**	**AT**	**AA**	**β (95% CI)**	**P**	**P**^**a**^
n	162	267	102			
**Anthropometric factors**						
Height (m)	1.38 ± 0.01	1.39 ± 0.01	1.40 ± 0.02	−0.003 (−0.01, 0.00)	0.148	0.594
Weight (kg)	42.3 ± 1.4	45.6 ± 1.1	50.1 ± 2.2	3.31 (1.52, 5.10)	**0.001**	0.397
BMI (kg/m^2^)	21.69 ± 0.48	22.89 ± 0.36	24.51 ± 0.64	1.32 (0.59, 2.05)	**0.001**	--
BMI z-score	1.48 ± 0.16	1.86 ± 0.13	2.32 ± 0.22	0.45 (0.19, 0.71)	**0.001**	--
Waist circumference (cm)	70.58 ± 1.39	72.85 ± 0.99	77.46 ± 1.71	3.35 (0.61, 6.10)	**0.017**	0.487
**Clinical and metabolic biomarkers**						
Systolic BP (mm Hg)	104 ± 1	104 ± 1	107 ± 1	1.14 (−0.71, 2.99)	0.227	0.466
Diastolic BP (mm Hg)	63 ± 1	65 ± 1	66 ± 1	1.24 (−0.26, 2.74)	0.106	0.888
Glucose (mg/dl)	85 ± 1	84 ± 0	84 ± 1	−0.47 (−1.34, 0.39)	0.286	0.143
Insulin (mU/l)	8.82 ± 0.64	8.73 ± 0.43	9.78 ± 0.71	0.02 (−0.01, 0.05)	0.253	0.225
HOMA-IR	1.89 ± 0.15	1.85 ± 0.10	2.07 ± 0.16	0.02 (−0.02, 0.05)	0.345	0.181
Triacylglycerols (mg/dl)	67 ± 3	64 ± 2	68 ± 4	0.07 (−3.90, 4.03)	0.974	0.216
ApoAI (mg/dl)	142 ± 2	141 ± 2	135 ± 3	−3.01 (−6.36, 0.34)	0.079	0.457
Cholesterol (mg/dl)	169 ± 3	166 ± 2	170 ± 2	0.002 (−0.01, 0.01)	0.721	0.540
HDL-c (mg/dl)	57 ± 1	57 ± 1	55 ± 1	−0.94 (−2.76, 0.89)	0.316	0.509
Adiponectin (mg/l)	25.89 ± 0.95	25.13 ± 0.74	24.16 ± 1.10	−0.72 (−2.18, 0.73)	0.331	0.934
Resistin (μg/l)	10.33 ± 0.44	10.81 ± 0.35	11.43 ± 0.55	0.55 (−0.15, 1.25)	0.125	0.384
Leptin (μg/l)	13.57 ± 1.23	14.37 ± 0.83	16.29 ± 1.52	1.38 (−0.43, 3.18)	0.135	0.149
**Inflammatory biomarkers**						
C-reactive protein (mg/l)	2.08 ± 0.29	2.21 ± 0.23	2.77 ± 0.53	0.37 (−0.14, 0.88)	0.153	0.743
IL-6 (ng/l)	5.61 ± 0.90	6.00 ± 0.69	6.19 ± 1.05	0.42 (−0.96, 1.80)	0.550	0.759
IL-8 (ng/l)	1.86 ± 0.16	1.95 ± 0.15	1.83 ± 0.21	0.03 (−0.25, 0.30)	0.853	0.783
TNF-α (ng/l)	3.53 ± 0.16	3.66 ± 0.13	3.33 ± 0.20	−0.02 (−0.27, 0.23)	0.875	0.308
**Cardiovascular disease risk biomarkers**						
MMP-9 (μg/l)	80.39 ± 4.35	83.62 ± 3.70	92.82 ± 6.05	0.03 (0.00, 0.07)	**0.049**	**0.048**
MPO (μg/l)	17.56 ± 2.06	17.84 ± 1.61	17.65 ± 1.88	0.29 (−2.80, 3.38)	0.853	0.636
sE-Selectin (μg/l)	26.34 ± 1.14	28.22 ± 1.05	26.64 ± 1.40	0.66 (−1.25, 2.56)	0.500	0.711
sICAM-1 (mg/l)	0.162 ± 0.005	0.168 ± 0.004	0.158 ± 0.006	0.001 (−0.01, 0.01)	0.997	0.645
Active PAI-1 (μg/l)	8.35 ± 0.58	8.61 ± 0.52	10.35 ± 0.97	0.85 (−0.19, 1.89)	0.109	0.946
Total PAI-1 (μg/l)	23.22 ± 1.34	22.21 ± 0.92	26.84 ± 2.09	0.02 (−0.02, 0.06)	0.359	0.994

CI: Confidence interval; BMI: body mass index; BP: blood pressure; HOMA-IR: homeostasis model assessment for insulin resistance; HDL-c: high-density lipoprotein cholesterol; IL: interleukin; TNF-α: tumour necrosis factor alpha; MMP-9: matrix metalloproteinase-9; MPO: myeloperoxidase; sICAM-1: soluble intracellular adhesion molecule-1, PAI-1: plasminogen activator inhibitor. β Coefficients represent the change in absolute traits values of each additional risk allele. General linear models were used to examine associations, P adjusted by age and sex, P^a^ adjusted by age, sex, and BMI.

Table 4 shows the association of the rs8061518 with obesity relate-traits. Genotype with the minor allele of this variant was negatively associated with leptin after adjustment for age, sex and BMI [β = −1.38 μg/l (95% CI: -2.70, -0.06)] (p = 0.041).

**Table 4:** Association of rs8061518 with anthropometric, clinical, inflammation and CVD risk biomarkers in children

**Biomarkers**	**AA**	**AG**	**GG**	**β (95% CI)**	**P**	***P***^***a***^
N	254	215	54			
**Anthropometric factors**						
Height (m)	1.39 ± 0.01	1.40 ± 0.01	1.39 ± 0.02	−0.001 (−0.01, 0.00)	0.613	0.107
Weight (kg)	46.8 ± 1.2	44.9 ± 1.3	42.6 ± 2.4	**−2.44 (−4.35, -0.54)**	**0.012**	0.130
BMI (kg/m^2^)	23.70 ± 0.38	22.33 ± 0.42	21.48 ± 0.81	**−1.27 (−2.05, -0.50)**	**0.001**	--
BMI z-score	2.16 ± 0.14	1.61 ± 0.14	1.36 ± 0.30	**−0.45 (−0.72, -0.17)**	**0.001**	--
Waist circumference (cm)	74.74 ± 1.07	71.99 ± 1.18	71.10 ± 2.25	−1.70 (−4.63, 1.22)	0.254	0.534
**Clinical and metabolic biomarkers**						
Systolic BP (mm Hg)	106 ± 1	103 ± 1	104 ± 2	−1.74 (−3.69, 0.21)	0.080	0.758
Diastolic BP (mm Hg)	66 ± 1	64 ± 1	61 ± 1	**−2.23 (−3.80, -0.65)**	**0.006**	0.083
Glucose (mg/dl)	85 ± 0	84 ± 0	85 ± 1	−0.11 (−1.02, 0.80)	0.816	0.836
Insulin (mU/l)	9.52 ± 0.48	8.56 ± 0.48	8.17 ± 1.13	**−0.05 (−0.09, -0.02)**	**0.003**	0.153
HOMA-IR	2.02 ± 0.10	1.80 ± 0.11	1.79 ± 0.30	**−0.05 (−0.09, -0.02)**	**0.003**	0.153
Triacylglycerols (mg/dl)	68 ± 2	63 ± 2	66 ± 6	−2.33 (−6.52, 1.86)	0.276	0.995
ApoAI (mg/dl)	141 ± 2	137 ± 2	145 ± 4	0.15 (−3.40, 3.71)	0.932	0.330
Cholesterol (mg/dl)	166 ± 2	168 ± 2	172 ± 4	0.01 (−0.004, 0.02)	0.223	0.287
HDL-c (mg/dl)	57 ± 1	56 ± 1	58 ± 2	0.07 (−1.87, 2.01)	0.945	0.107
Adiponectin (mg/l)	25.46 ± 0.77	23.92 ± 0.73	27.90 ± 1.81	0.38 (−1.15, 1.91)	0.628	0.741
Resistin (μg/l)	11.34 ± 0.40	10.30 ± 0.33	10.37 ± 0.72	−0.68 (−1.42, 0.06)	0.074	0.227
Leptin (μg/l)	16.98 ± 1.02	12.57 ± 0.85	11.21 ± 1.70	**−3.55 (−5.43, -1.66)**	**<0.001**	**0.041**
**Inflammatory biomarkers**						
C-reactive protein (mg/l)	2.50 ± 0.26	2.26 ± 0.31	1.70 ± 0.30	−0.34 (−0.89, 0.20)	0.217	0.784
IL-6 (ng/l)	5.62 ± 0.64	6.56 ± 0.90	4.71 ± 0.91	0.09 (−1.36, 1.53)	0.905	0.705
IL-8 (ng/l)	1.94 ± 0.14	1.98 ± 0.17	1.57 ± 0.19	−0.10 (−0.39, 0.19)	0.495	0.804
TNFα (ng/l)	3.62 ± 0.14	3.51 ± 0.14	3.60 ± 0.27	−0.05 (−0.32, 0.22)	0.711	0.696
**Cardiovascular disease risk biomarkers**						
MMP-9 (μg/l)	82.62 ± 3.42	86.21 ± 4.14	84.12 ±8.05	0.01 (−0.03, 0.04)	0.764	0.754
MPO (μg/l)	19.19 ± 1.68	16.65 ± 1.44	16.55 ± 4.26	−1.63 (−4.94, 1.68)	0.336	0.712
sE-Selectin (μg/l)	28.19 ± 0.98	26.13 ± 1.08	26.61 ± 2.34	−1.09 (−3.13, 0.95)	0.296	0.924
sICAM-1 (mg/l)	0.165 ± 0.004	0.168 ± 0.005	0.155 ± 0.007	−0.002 (−0.01, 0.01)	0.605	0.923
Active PAI-1 (μg/l)	9.81 ± 0.62	8.45 ± 0.49	6.16 ± 0.63	**−1.71 (−2.80, -0.62)**	**0.002**	0.067
Total PAI-1 (μg/l)	24.74 ± 1.11	22.56 ± 1.16	20.66 ± 1.85	−0.04 (−0.08, 0.003)	0.073	0.350

CI: Confidence Interval; BMI: body mass index; BP: blood pressure; HOMA-IR: homeostasis model assessment for insulin resistance; HDL-c: High-density lipoprotein-cholesterol; IL: interleukin; TNFα: tumour necrosis factor alpha; MMP-9: matrix metalloproteinase-9; MPO: myeloperoxidase; sICAM-1: soluble intracellular adhesion molecule-1; PAI-1: plasminogen activator inhibitor-1. β coefficients represent the change in absolute traits values of each additional risk allele. General linear models were used to examine associations, P adjusted by age and sex, P^a^ adjusted by age, sex, and BMI.

### Linkage disequilibrium and haplotype block analysis

**Figure 1:**
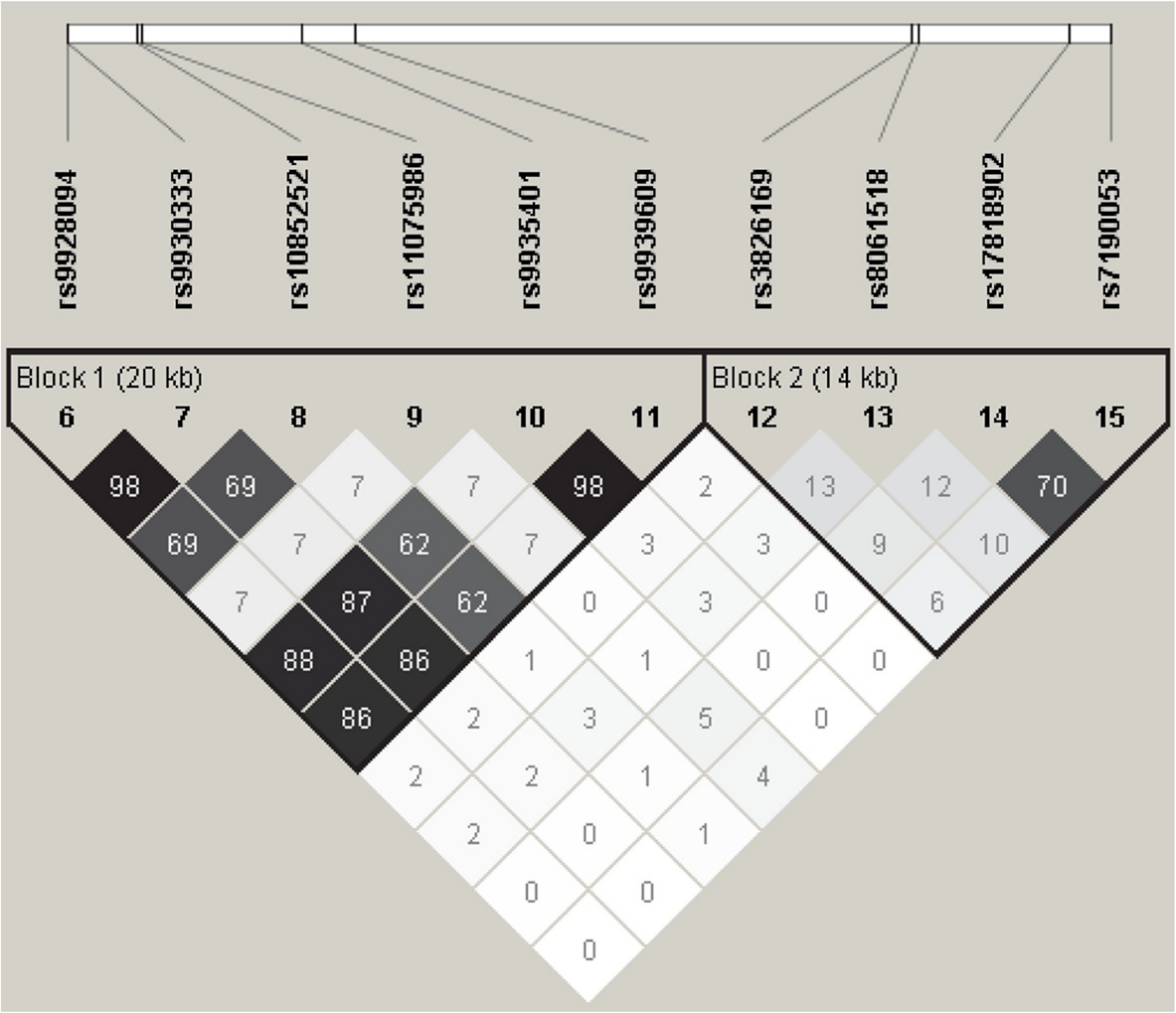
**Linkage disequilibrium structure in terms of r**^**2 **^**of 10 SNPs genotyped, representing two well-defined haplo-blocks.**

Figure 1 shows the LD of two well-defined haplo-blocks. In this figure, we can observe the LD between the 5 SNPs significantly associated with obesity. rs9928094, rs9930333, rs9935401 and rs9939609 are in LD between each other, while rs8061518 is in LD with no one of the above. Table 5 shows the total population haplotype blocks frequency as well as cases and controls separately. The haplo-block 1 involves the SNPs rs9928094, rs9930333, rs10852521, rs11075986, rs9935401 and rs9939609. The overall p value of χ^2^ for association with obesity for this haplo-block was 8.72 × 10^-4^. The risk for obesity associated with the haplotype GGCCAA (which include the risk allele of rs9939609-A and the risk alleles of the rs9928094-G, rs9930333-G and rs9935401-A all of them in LD with the former) was 1.26 (95% CI: 1.64 - 2.14) (p = 5.11 × 10^-5^). The haplo-block 2 involves the SNPs rs3826169, rs8061518, rs17818902 and rs7190053. The overall p value of χ^2^ for association with obesity for this haplo-block was 0.026. The risk for obesity associated with the haplotype TGTC (the only one that include the rs8061518-G allele, that showed a negative association with obesity) was 0.65 (95% CI: 0.47 – 0.92) (p = 0.002).

**Table 5:** **Association analysis of obesity with *FTO *** **haplotypes combinations**

**Haplotypes**	**Frequency**				
	**All**	**Case**	**Control**	**OR (95% CI)**	**P**
**Block 1**					
ATTCGT	0.437	0.401	0.481	Reference	
GGCCAA	0.425	0.483	0.355	**1.26 (1.64 - 2.14)**	**5.11 × 10**^**-5**^
ATCGGT	0.080	0.073	0.087	0.99 (0.63 - 1.57 )	0.413
GGCCGT	0.025	0.019	0.031	0.65 (0.29 - 1.45)	0.133
**Block 2**					
CATC	0.223	0.237	0.207	Reference	
TGTC	0.297	0.258	0.345	**0.65 (0.47-0.92)**	**0.002**
TATC	0.212	0.225	0.196	1.02 (0.71-1.45)	0.231
TAGT	0.179	0.193	0.163	1.02 (0.70-1.51)	0.217
TAGC	0.053	0.063	0.041	1.29 (0.70 - 2.38)	0.143

Block 1 involves SNPs: rs9928094, rs9930333, rs10852521, rs11075986, rs9935401, and rs9939609, Overall p value: 8.72 × 10^-4^. Block 2 involves SNPs: rs3826169, rs8061518, rs17818902, and rs7190053. Overall p value: 0.026.

OR: odds ratio; CI: confidence interval.

## Discussion

In the present study, where we studied 52 *FTO* variants spanning the whole gene, we validate the positive association of rs9939609 with obesity in Spanish children. We found three other SNPs (rs9935401, rs9928094, and 9930333), also localized in the first intron of the gene positively associated with obesity. Additionally, we observed for the first time, the association of rs8061518, localized in the third intron of the gene, with a reduced risk of obesity and low plasma leptin concentrations.

Our results are in concordance with many studies, which have shown the association of the rs9939609 with adulthood and childhood obesity [3,19], as well as other SNPs in LD with it, such as the rs9935401, rs9928094 and rs9930333 [16,20,21]. Several studies among European descent population have demonstrated the association of variant rs9939609 with anthropometric parameters, such as BMI, weight, WC [22] and skin folds [23]. Interestingly, the association with BMI found in carriers of haplotype GGCCAA (which include the risk alleles of the SNPs positively associated with obesity) could be useful for the early identification of inherited susceptibility to weight-gain beginning during the childhood, with a higher sensitivity than considering the effect of a single marker.

Among the large number of phenotypes measured in this study, we did not find any significant association with *FTO* variants located in the first intron after additional adjustment for BMI, except in the case of MMP-9, a well-known biomarker of CVD risk [24], which has shown an association not only with rs9939609 but also with rs9928094 and rs9930333. The association of rs9939609 with CVD markers are opposed in the scientific literature. Whereas some have shown significant association with low HDL-c [25,26] and high triacylglycerols [25], others [27,28] have not found any association. Additionally, there are few studies that link CVD and *FTO* gene [29,30], and to our knowledge, there are no studies that have included CVD risk biomarkers as MPO, sE-selectin, sICAM-1, PAI-1 and MMP-9 as we did here. Some other studies have shown the association of rs9939609 with inflammatory biomarkers as in the case of Timpson *et al.*[31] and Fisher *et al.*[32]; both found a positive association with CRP. Zimmerman *et al.*[33] included other biomarkers, such as IL-6, IL-1β, IL-10, IL-18 or TNF α, and found no significant association in line with our findings. The fact that the majority of studies has failed to associate the *FTO* variants with obesity related trait, indicates that this gene is in a very close relationship with obesity and fat mass. The potential effect on the obesity-related traits seems indirect and dependent on BMI changes.

The association of the variant rs9939609 with MS has been shown in European [25] and mixed population [34]. Furthermore, rs1421085 which is highly correlated (LD r^2^ = 0.931) [3] with rs9939609, was associated with MS in an independent study [35]. On the contrary, rs9939609 was not associated with MS in Japanese [36] or Indian [37] population. As we discussed previously, some studies showed an association with cardiovascular markers, also related to MS, whereas others did not. The same is true for glucose metabolism and BP; some studies found a significant association with glucose and insulin [38] and others, in concordance with our work, did not [26-28]. In the case of BP, neither Freathy *et al.*[25] nor Kring *et al.*[26] observed an association; however, Pausova *et al.*[39] did otherwise with systolic BP in adolescents. Moreover, a recent genome-wide scan identified an association between rs9930333 (SNP in LD with rs9939609) and BP in adolescents [40]. In our study population, the percentage of MS in the obese group was 9.8%, and we did not observe association of any SNPs with MS (data not shown). These results could be due to the small size of the study population as it has been reported that sample sizes of at least 12000 are required to observe association between *FTO* genotype and metabolic traits that are secondary to changes in BMI [25]. In addition, the lack of association found in our study might also be due to the age of our population (mean of 9.6 years), because it has been demonstrated that rs9939609 showed changes in its association with BMI as children grow up and enter into youth. One study showed no association at age of 4 years, but this association became increasingly stronger at ages 7–11 years [41]; other study, observed a biphasic change in the associations of this variant with BMI, strengthening with age up to a peak at age 20 years, and then weakening with increasing adult age [42].

Beyond the replication of association found in SNPs within the first intron, we interestingly identified a novel SNP (rs8061518), localized in the third intron of the gene about 40–60 kb away from the originally reported SNPs in the first intron, which was associated with reduced risk of obesity. This SNP was also negatively associated with some MS traits, leptin and aPAI-1. Although our results should be validated in larger independent studies, we used the publicly available GIANT consortium published data [43], and we found that the rs8061518 was associated with BMI with a p = 0.0149 in a population of 123844 European descent adults, and the association effect was in the same direction that we observed in our population. Only one previous study in Sorb population [44] has demonstrated that other SNPs in the same region of the gene were associated with reduced risk of obesity, among them the strongest effect on BMI values was associated with rs17818902. However, in the present study we did not confirm this association, in concordance with Dlouha *et al.* who genotyped this SNP in a group of 2559 unrelated European descent population [45]. In addition, no association of this variant with BMI was found in the GIANT consortium (p = 0.206). One possible explanation of this is that the rs17818902 represents a second mutational event segregating in the Sorbian population.

The haplotype analysis shows that a unique haplotype TGTC (haplo-block 2) with the minor allele of the rs8061518 exhibited a decreased risk for obesity. These results indicate that there may be other regions beyond the intron 1 in the *FTO* gene associated with being lean or obese that needs to be studied.

The association of the *FTO* gene with leptin has also been studied. Labayen *et al.*[9], Rutters *et al.*[14], and Zimmerman *et al.*[33] found a positive association of the rs9939609 with leptin, whereas Do *et al.*[23] did it with the rs17817449; however, this association was abolished after adjusting for BMI. In our population, we did not observe an association in the case of the rs9939609, but interestingly we found a negative association with rs8061518 that remains significant after adjustment by sex, age and BMI. Leptin, a hormone produced mainly by the white adipose tissue, is clearly associated with the total amount of body fat and secondly with BMI. It participates in the regulation of a range of biological functions and process including energy intake and expenditure, neuroendocrine system, autonomic function, reproduction, and glucose homeostasis [46,47]. As this hormone is involved in three different pathways [47], its mechanisms of action act through different signalling pathways. Studies in animals [48] and humans [49] have demonstrated that it can exert its action independently of obesity. Then, it might be the case in the association of this adipokine with the rs8061518; however, further studies are needed to clarify this aspect.

Our study has several strengths and limitations to highlight. The strengths are the high quantity of biomarkers measured in the children, the genotyping of several SNPs along the *FTO* gene and the way of SNPs election. We selected part of the SNPs from a group with a MAF higher than 0.05 in the European descent population from the NCBI database to make an association case–control study as complementary strategy to GWAS in the identification of gene variants associated with obesity and its comorbidities. The limitations are first, the sample size of the current investigation is relatively small for a genetic association with metabolic traits. Second, there are a number of environmental factors known to influence body weight in children, which were not taken into account in this study. Furthermore, information on body composition, nutrition and physical activity, which are strongly associated factors with body-weight regulation, was not available. Fat composition data could be valuable for the adjustment of the association of rs8061518 with leptin in order to clarify the role of the genetic variant on obesity.

## Conclusion

We confirmed the previously reported association of genetic variability in intron 1 of the *FTO* gene with the risk of obesity and without association with other related traits of inflammation and CVD risk biomarkers. We also have observed strong statistical evidence for an association of rs8061518 in intron 3 of the gene with decreased risk of obesity and low concentration of leptin. Additionally, both studied haplo-blocks, that included the significant associated SNPs, were also associated with the risk of obesity. Our findings provide qualified support for the notion that a detailed examination of the third intron region of the *FTO* gene in larger independent studies should provide valuable clues to the molecular mechanisms by which sequence variation in this region affects clinical phenotypes.

## Abbreviations

ApoA1: Apolipoprotein A1; ApoB: Apolipoprotein B; BMI: Body mass index; BP: Blood pressure; CI: Confidence interval; CV: Coefficient of variation; CVD: Cardiovascular diseases; FTO: Fat mass and obesity associated; HDL-c: High-density lipoprotein-cholesterol; HOMA-IR: Homeostatic model assessment of insulin resistance; IL: Interleukin; LD: Linkage disequilibrium; LDL-c: Density lipoprotein-cholesterol; MAF: Minor allele frequency; MMP-9: Matrix metalloproteinase-9; MPO: Myeloperoxidase; MS: Metabolic syndrome; OR: Odds ratio; PAI-1: Plasminogen activator inhibitor-1; sE-selectin: Soluble endothelial selectin; sICAM-1: Soluble intercellular adhesion molecule 1; SNP: Single nucleotide polymorphism; TNF-α: Tumour necrosis factor alpha; WC: Waist circumference.

## Competing interests

The authors declare that they have no competing interests.

## Authors’ contributions

Conception and design of the study: AG, CMA. Collection of the children’s and acquisition of the data: MG-C, RL, RT, RC. Biomarkers analysis: JO. Genotyping analysis: DF-O. Statistical analysis: JO, AIR, DF-O. Interpretation of the data and drafting the manuscript: JO, CMA. Critical revision of the manuscript: JO, AG, CMA. All authors have read and approved the final version of the manuscript.

## Pre-publication history

The pre-publication history for this paper can be accessed here:

http://www.biomedcentral.com/1471-2350/14/123/prepub

## Supplementary Material

Additional file 1: Table S1 Description of the genotyped polymorphisms. **Table S2**. Association of rs9928094 with biomarkers of inflammation and cardiovascular disease risk in obese children. **Table S3**. Association of rs9930333 with biomarkers of inflammation and cardiovascular disease risk in obese children. **Table S4**. Association of rs9935401 with biomarkers of inflammation and cardiovascular disease risk in obese children. Click here for file
