# Supplementary material for: Influence of FTO variants on obesity, inflammation and cardiovascular disease risk biomarkers in Spanish children: a case–control multicentre study
Source: BMC Med Genet. 2013 Dec 1;14:123. doi: 10.1186/1471-2350-14-123 (PMC3866940; doi:10.1186/1471-2350-14-123)
Supplement: Additional file 1: Table S1 — Description of the genotyped polymorphisms. Table S2. Association of rs9928094 with biomarkers of inflammation and cardiovascular disease risk in obese children. Table S3. Association of rs9930333 with biomarkers of inflammation and cardiovascular disease risk in obese children. Table S4. Association of rs9935401 with biomarkers of inflammation and cardiovascular disease risk in obese children. [file 1471-2350-14-123-S1.pdf]

Additional files

Additional Table 1 Description of the genotyped polymorphisms.

Polymorphism	Position	MAF	Alleles (M/m)	HWE P	*Call rate* (%)
rs9939609	Intron 1	0.444	T/A	0.682	99.4
rs9928094	Intron 1	0.462	A/G	0.789	99.8
rs9930333	Intron 1	0.463	T/G	0.687	99.4
rs9935401	Intron 1	0.439	G/A	1	99.3
rs10852521	Intron 1	0.446	C/T	0.606	99.8
rs1861868	Intron 1	0.466	A/G	0.797	99.8
rs7205986	Intron 1	0.084	A/G	0.246	99.8
rs11643744	Intron 1	0.267	A/G	0.256	99.8
rs7203521	Intron 1	0.383	A/G	1	100
rs12445162	Intron 1	0.075	G/A	0.681	100
rs11075986	Intron 1	0.084	C/G	0.201	100
rs17818902	Intron 3	0.245	T/G	0.855	99.8
rs3826169	Intron 3	0.228	T/C	1	100
rs8061518	Intron 3	0.309	A/G	0.889	97.9
rs7190053	Intron 3	0.183	C/T	1	99.1
rs2111114	Intron 4	0.212	A/G	0.843	99.8
rs8044353	Intron 4	0.053	G/A	1	99.8
rs10521303	Intron 5	0.439	C/A	0.069	99.1
rs1558756	Intron 6	0.444	C/T	0.119	100
rs16952623	Intron 6	0.132	T/C	0.388	99.4
rs7194336	Intron 7	0.441	G/T	0.199	100

rs9929152	Intron 7	0.277	A/G	**0.040**	99.6
rs16952649	Intron 7	0.122	C/T	**0.008**	100
rs10852525	Intron 7	0.106	G/A	0.159	99.1
rs8053966	Intron 7	0.184	T/C	**0.007**	99.6
rs2111113	Intron 7	0.074	G/C	0.330	99.6
rs16952624	Exon 7	0.001	C/T	1	99.6
rs1136002	Intron 8	0.317	T/C	0.462	100
rs2075202	Intron 8	0.025	T/G	1	100
rs2540781	Intron 8	0.128	C/A	0.345	100
rs12932373	Intron 8	0.138	C/T	0.589	99.4
rs1008400	Intron 8	0.459	C/T	0.153	99.6
rs1125337	Intron 8	0.450	A/T	**0.027**	99.8
rs17833492	Intron 8	0.313	C/A	0.466	99.6
rs7203181	Intron 8	0.339	C/A	0.780	100
rs7194907	Intron 8	0.479	T/C	0.091	98.9
rs2689248	Intron 8	0.480	C/A	0.368	99.4
rs13334214	Intron 8	0.201	C/T	0.228	98.3
rs1558687	Intron 8	0.242	C/T	0.864	99.4
rs4784351	Intron 8	0.270	A/G	0.742	97.6
rs12596638	Intron 8	0.136	G/A	1	99.8
rs8056299	Intron 8	0.429	A/G	0.696	99.8
rs7194243	Intron 8	0.219	C/T	0.337	100
rs6499656	Intron 8	0.130	G/C	0.356	100
rs7199716	Intron 8	0.394	C/T	0.893	99.8
rs8054364	Intron 8	0.146	C/G	**0.040**	99.8

rs8049235	Intron 8	0.393	G/A	0.285	99.6
rs17225435	Intron 8	0.115	A/G	1	100
rs7200579	Intron 8	0.060	C/G	0.536	100
rs697771	Intron 8	0.427	C/T	0.362	100
rs7191513	Intron 8	0.424	G/A	0.241	100
rs8049933	Intron 8	0.108	C/T	0.284	99.3

HWE P: Hardy-Weinberg equilibrium (p for the control group); FAM: minor allele frequency.

Additional Table 2 Association of rs9928094 with biomarkers of inflammation and cardiovascular disease risk in obese children.

	AA	AG	GG	β (95%CI)	P
**n**	155	263	115		
**Anthropometric factors**					
Height (m)	1.38 ± 0.01	1.39 ± 0.01	1.39 ± 0.01	-0.003 (-0.01, 0.00)	0.167
Weight (kg)	42.2 ± 1.4	45.9 ± 1.1	48.8 ± 2.0	3.14 (1.37, 4.91)	**0.001**
BMI (kg/m^2)	21.55 ± 0.49	23.02 ± 0.36	24.12 ± 0.61	1.26 (0.53, 1.98)	**0.001**
BMI z-score	1.43 ± 0.17	1.88 ± 0.13	2.25 ± 0.20	0.44 (0.18, 0.69)	**0.001**
Waist circumference (cm)	70.43 ± 1.40	73.16 ± 1.01	76.45 ± 1.61	2.77 (0.03, 5.51)	**0.048**
**Clinical and metabolic biomarkers**					
Systolic BP (mm Hg)	104 ± 1	105 ± 1	105 ± 1	0.90 (-0.91, 2.72)	0.330
Diastolic BP (mm Hg)	63 ± 1	65 ± 1	65 ± 1	0.81 (-0.67, 2.28)	0.285
Glucose (mg/dl)	85 ± 1	84 ± 0	84 ± 1	-0.37 (-1.22, 0.49)	0.399
Insulin (mU/l)	8.81 ± 0.66	8.86 ± 0.43	9.35 ± 0.65	0.02 (-0.02, 0.05)	0.349
HOMA-IR	1.88 ± 0.15	1.87 ± 0.10	1.98 ± 0.14	0.01 (-0.02, 0.05)	0.443
Triacylglycerol (mg/dl)	68 ± 3	63 ± 2	68 ± 3	-0.64 (-0.45, 3.27)	0.748

Apo-AI (mg/dl)	143 ± 2	140 ± 2	136 ± 3	-3.41 (-6.72, -0.09)	**0.044**
Cholesterol (mg/dl)	169 ± 3	166 ± 2	168 ± 2	-0.001 (-0.01, 0.01)	0.811
HDL-c (mg/dl)	58 ± 1	57 ± 1	55 ± 1	-0.84 (-2.65, 0.96)	0.360
Adiponectin (mg/l)	25.94 ± 0.99	25.15 ± 0.73	24.22 ± 1.06	-0.50 (-1.93, 0.93)	0.496
Resistin (µg/l)	10.28 ± 0.46	10.91 ± 0.35	11.15 ± 0.51	0.50 (-0.19, 1.19)	0.158
Leptin (µg/l)	13.69 ± 1.29	14.50 ± 0.84	15.55 ± 1.38	1.00 (-0.78, 2.78)	0.273
**Inflammatory biomarkers**					
C-reactive protein (mg/l)	2.02 ± 0.30	2.31 ± 0.24	2.59 ± 0.48	0.31 (-0.19, 0.81)	0.228
IL-6 (ng/l)	5.73 ± 0.93	5.66 ± 0.62	6.67 ± 1.21	0.53 (-0.83, 1.89)	0.448
IL-8 (ng/l)	1.87 ± 0.16	1.90 ± 0.14	1.95 ± 0.22	0.06 (-0.21, 0.34)	0.645
TNFα (ng/l)	3.58 ± 0.17	3.69 ± 0.13	3.30 ± 0.18	-0.07 (-0.32, 0.18)	0.568
**Cardiovascular disease risk biomarkers**					
MMP-9 (µg/l)	82.36 ± 4.57	80.95 ± 3.67	94.46 ± 5.61	0.04 (0.003, 0.07)	**0.034**
MPO (µg/l)	17.77 ± 2.16	17.67 ± 1.61	17.63 ± 1.75	0.14 (-2.91, 3.19)	0.929
sE-Selectin (µg/l)	26.06 ± 1.17	28.43 ± 1.06	26.38 ± 1.33	0.61 (-1.27, 2.50)	0.525
sICAM-1 (mg/l)	0.162 ± 0.005	0.168 ± 0.005	0.160 ± 0.005	0.004 (-0.01, 0.01)	0.993

Active PAI-1 (µg/l)	8.39 ± 0.60	8.64 ± 0.52	10.04 ± 0.88	0.80 (-0.22, 1.82)	0.127
Total PAI-1 (µg/l)	23.04 ± 1.34	22.22 ± 0.94	26.72 ± 1.90	0.02 (-0.01, 0.06)	0.223

CI: Confidence interval; BMI: body mass index; BP: blood pressure; HOMA-IR: homeostasis model assessment for insulin resistance; HDL-c: high-density lipoprotein cholesterol; IL: interleukin; TNF-α: tumor necrosis factor alpha; MMP-9: metalloproteinase-9; MPO: myeloperoxidase; sICAM-1: soluble intracellular adhesion molecule-1, PAI-1: plasminogen activator inhibitor. β Coefficients represent the change in absolute traits values of each additional risk allele. General linear models were used to examine associations, P adjusted by age and sex.

Additional Table 3 Association of rs9930333 with biomarkers of inflammation and cardiovascular disease risk in obese children.

	**TT**	**TG**	**GG**	**β (95% CI)**	**P**
**n**	154	262	115		
**Anthropometric factors**					
Height (m)	1.38 ± 0.01	1.39 ± 0.01	1.39 ± 0.01	-0.003 (-0.01, 0.00)	0.192
Weight (kg)	42.4 ± 1.4	46.0 ± 1.1	48.5 ± 2.0	3.07 (1.31, 4.84)	**0.001**
BMI (kg/m^2)	21.60 ± 0.49	23.09 ± 0.36	24.03 ± 0.61	1.23 (0.52, 1.96)	**0.001**
BMI z-score	1.44 ± 0.17	1.90 ± 0.13	2.23 ± 0.20	0.43 (0.18, 0.69)	**0.001**
Waist circumference (cm)	70.54 ± 1.41	73.31 ± 1.02	76.30 ± 1.61	2.73 (-0.003, 5.47)	0.051
**Clinical and metabolic biomarkers**					
Systolic BP (mm Hg)	104 ± 1	105 ± 1	105 ± 1	0.88 (-0.93, 2.69)	0.339
Diastolic BP (mm Hg)	63 ± 1	65 ± 1	65 ± 1	0.83 (-0.65, 2.30)	0.272
Glucose (mg/dl)	85 ± 1	84 ± 0	84 ± 1	-0.37 (-1.22, 0.49)	0.402
Insulin (mU/l)	8.83 ± 0.67	8.89 ± 0.43	9.31 ± 0.65	0.02 (-0.02, 0.05)	0.400
HOMA-IR	1.89 ± 0.15	1.88 ± 0.10	1.97 ± 0.14	0.01 (-0.02, 0.05)	0.499
Triacylglycerols (mg/dl)	68 ± 3	63 ± 2	68 ± 3	-0.42 (-4.32, 3.48)	0.034

Apo-AI (mg/dl)	143 ± 2	141 ± 2	135 ± 3	-3.59 (-6.90, -0.29)	**0.033**
Cholesterol (mg/dl)	169 ± 3	167 ± 2	168 ± 2	-0.002 (-0.01, 0.01)	0.728
HDL-c (mg/dl)	57 ± 1	57 ± 1	55 ± 1	-0.97 (-2.77, 0.83)	0.293
Adiponectin (mg/l)	25.77 ± 0.98	25.37 ± 0.75	23.78 ± 1.01	-0.73 (-2.16, 0.70)	0.319
Resistin (µg/l)	10.28 ± 0.46	10.95 ± 0.35	11.10 ± 0.51	0.45 (-0.23, 1.14)	0.198
Leptin (µg/l)	13.76 ± 1.29	14.56 ± 0.84	15.56 ± 1.38	0.98 (-0.80, 2.75)	0.283
**Inflammatory biomarkers**					
C-reactive protein (mg/l)	2.03 ± 0.30	2.31 ± 0.24	2.60 ± 0.48	0.32 (-0.18, 0.82)	0.371
IL-6 (ng/l)	5.74 ± 0.93	5.67 ± 0.62	6.68 ± 1.21	0.51 (-0.84, 1.87)	0.461
IL-8 (ng/l)	1.86 ± 0.16	1.90 ± 0.15	1.95 ± 0.22	0.06 (-0.21, 0.33)	0.656
TNFα (ng/l)	3.57 ± 0.17	3.70 ± 0.13	3.32 ± 0.18	-0.08 (-0.32, 0.17)	0.552
**Cardiovascular disease risk biomarkers**					
MMP-9 (µg/l)	81.61 ± 4.54	80.95 ± 3.71	96.34 ± 5.60	0.04 (0.006, 0.07)	**0.021**
MPO (µg/l)	17.65 ± 2.17	17.78 ± 1.62	17.57 ± 1.75	0.07 (-2.97, 3.12)	0.962
sE-Selectin (µg/l)	26.06 ± 1.17	28.43 ± 1.06	26.38 ± 1.33	0.56 (-1.33, 2.44)	0.562
sICAM-1 (mg/l)	0.162 ± 0.005	0.169 ± 0.005	0.159 ± 0.005	-0.000 (-0.01, 0.01)	0.953

Active PAI-1 (μg/l)	8.40 ± 0.60	8.69 ± 0.52	10.06 ± 0.88	0.79 (-0.23, 1.81)	0.129
Total PAI-1 (μg/l)	23.02 ± 1.35	22.13 ± 0.94	27.04 ± 1.90	0.03 (-0.01, 0.06)	0.187

CI: Confidence interval; BMI: body mass index; BP: blood pressure; HOMA-IR: homeostasis model assessment for insulin resistance; HDL-c: high-density lipoprotein cholesterol; IL: interleukin; TNF-α: tumor necrosis factor alpha; MMP-9: metalloproteinase-9; MPO: myeloperoxidase; sICAM-1: soluble intracellular adhesion molecule-1, PAI-1: plasminogen activator inhibitor. β Coefficients represent the change in absolute traits values of each additional risk allele. General linear models were used to examine associations, P adjusted by age and sex.

Additional Table 4 Association of rs9935401 with biomarkers of inflammation and cardiovascular disease risk in obese children.

	**GG**	**AG**	**AA**	**β (95%CI)**	**P**
**n**	167	261	102		
**Anthropometric factors**					
Height (m)	1.38 ± 0.01	1.39 ± 0.01	1.40 ± 0.02	-0.003 (-0.01, 0.001)	0.115
Weight (kg)	42.1 ± 1.4	45.9 ± 1.1	50.1 ± 2.2	3.44 (1.66, 5.22)	**<0.001**
BMI (kg/m^2)	21.56 ± 0.47	23.08 ± 0.37	24.51 ± 0.64	1.37 (0.65, 2.10)	**<0.001**
BMI z-score	1.42 ± 0.16	1.93 ± 0.13	2.32 ± 0.22	0.47 (0.22, 0.73)	**<0.001**
Waist circumference (cm)	70.29 ± 1.35	73.35 ± 1.01	77.46 ± 1.71	3.37 (0.62, 6.14)	**0.017**
**Clinical and metabolic biomarkers**					
Systolic BP (mm Hg)	104 ± 1	105 ± 1	107 ± 1	1.19 (-0.65, 3.02)	0.205
Diastolic BP (mm Hg)	63 ± 1	65 ± 1	66 ± 1	1.39 (-0.10, 2.88)	0.068
Glucose (mg/dl)	85 ± 1	84 ± 0	84 ± 1	-0.42 (-1.28, 0.44)	0.342
Insulin (mU/l)	8.78 ± 0.62	8.78 ± 0.44	9.78 ± 0.71	0.02 (-0.01, 0.05)	0.209
HOMA-IR	66 ± 3	64 ± 2	68 ± 4	0.34 (-3.61, 4.29)	0.866
Triacylglycerols (mg/dl)	1.88 ± 0.14	1.86 ± 0.10	2.07 ± 0.16	0.02 (-0.02, 0.05)	0.286

Apo-AI (mg/dl)	143 ± 2	141 ± 2	135 ±	-3.04 (-6.38, 0.31)	0.076
Cholesterol (mg/dl)	168 ± 2	166 ± 2	170 ± 2	0.003 (-0.01, 0.01)	0.584
HDL-c (mg/dl)	58 ± 1	57 ± 1	55 ± 1	-1.04 (-2.86, 0.79)	0.266
Adiponectin (mg/l)	26.09 ± 0.95	24.80 ± 0.73	24.16 ± 1.10	-0.67 (-2.11, 0.78)	0.366
Resistin (µg/l)	10.30 ± 0.43	10.85 ± 0.36	11.43 ± 0.55	0.59 (-0.10, 1.29)	0.096
Leptin (µg/l)	13.29 ± 1.20	14.68 ± 0.84	16.29 ± 1.52	1.51 (-0.28, 3.30)	0.100
**Inflammatory biomarkers**					
C-reactive protein (mg/l)	2.05 ± 0.29	2.29 ± 0.23	2.77 ± 0.53	0.38 (-0.12, 1.89)	0.140
IL-6 (ng/l)	5.56 ± 0.87	6.00 ± 0.70	6.19 ± 1.05	0.44 (-0.93, 1.82)	0.528
IL-8 (ng/l)	1.86 ± 0.15	1.96 ± 0.15	1.83 ± 0.21	0.03 (-0.24, 0.31)	0.819
TNFα (ng/l)	3.52 ± 0.16	3.69 ± 0.13	3.33 ± 0.20	-0.01 (-0.26, 0.24)	0.962
**Cardiovascular disease risk biomarkers**					
MMP-9 (µg/l)	82.52 ± 4.42	82.49 ± 3.73	92.82 ± 6.05	0.03 (-0.004, 0.07)	0.080
MPO (µg/l)	17.65 ± 2.02	17.92 ± 1.63	17.65 ± 1.88	0.27 (-2.81, 3.35)	0.865
sE-Selectin (µg/l)	26.18 ± 1.12	28.25 ± 1.07	26.64 ± 1.40	0.74 (-1.16, 2.64)	0.448
sICAM-1 (mg/l)	0.162 ± 0.005	0.169 ± 0.005	0.158 ± 0.006	0.0003 (-0.01, 0.01)	0.951

Active PAI-1 (µg/l)	8.41 ± 0.58	8.62 ± 0.52	10.35 ± 0.97	0.90 (-0.13, 1.93)	0.089
Total PAI-1 (µg/l)	23.26 ± 1.31	22.18 ± 0.92	26.84 ± 2.09	0.02 (-0.02, 0.06)	0.313

CI: Confidence interval; BMI: body mass index; BP: blood pressure; HOMA-IR: homeostasis model assessment for insulin resistance; HDL-c: high-density lipoprotein cholesterol; IL: interleukin; TNF-α: tumor necrosis factor alpha; MMP-9: metalloproteinase-9; MPO: myeloperoxidase; sICAM-1: soluble intracellular adhesion molecule-1, PAI-1: plasminogen activator inhibitor. β Coefficients represent the change in absolute traits values of each additional risk allele. General linear models were used to examine associations, P adjusted by age and sex.
